# Bismuth Quantum Dot (Bi QD)/Polydimethylsiloxane (PDMS) Nanocomposites with Self-Cleaning and Antibacterial Activity for Dental Applications

**DOI:** 10.3390/nano12213911

**Published:** 2022-11-05

**Authors:** Yingzi Hu, Zhiliang Xu, Yi Hu, Lanping Hu, You Zi, Mengke Wang, Xingmei Feng, Weichun Huang

**Affiliations:** 1 Department of Stomatology, Affiliated Hospital of Nantong University, 20 Xisi Road, Nantong 226001, China; 2 School of Chemistry and Chemical Engineering, Nantong University, Nantong 226019, China

**Keywords:** bismuth, quantum dots, nanocomposite, self-cleaning, antibacterial

## Abstract

In the oral microenvironment, bacteria colonies are easily aggregated on the tooth-restoration surface, in the manner of a biofilm, which usually consists of heterogeneous structures containing clusters of a variety of bacteria embedded in an extracellular matrix, leading to serious recurrent caries. In this contribution, zero-dimensional (0D) bismuth (Bi) quantum dots (QDs) synthesized by a facile solvothermal method were directly employed to fabricate a Bi QD/polydimethylsiloxane (PDMS)-modified tooth by simple curing treatment. The result demonstrates that the as-fabricated Bi QD/PDMS-modified tooth at 37 °C for 120 min not only showed significantly improved hydrophobic performance with a water contact angle of 103° and 115° on the tooth root and tooth crown, respectively, compared to that (~20° on the tooth root, and ~5° on the tooth crown) of the pristine tooth, but also exhibited excellent antibacterial activity against *S. mutans*, superior biocompatibility, and biosafety. In addition, due to the highly photothermal effect of Bi QDs, the antibacterial activity of the as-fabricated Bi QD/PDMS-modified tooth could be further enhanced under illumination, even at a very low power density (12 mW cm^−2^). Due to the facile fabrication, excellent hydrophobicity, superior antibacterial activity, and biocompatibility and biosafety of the Bi QD/PDMS-modified tooth, it is envisioned that the Bi QD/PDMS-modified tooth with a fascinating self-cleaning and antibacterial performance can pave the way to new designs of versatile multifunctional nanocomposites to prevent secondary caries in the application of dental restoration.

## 1. Introduction

Dental caries, a prevalent oral disease, is a very complicated interaction between fermentable carbohydrates and acid-producing bacteria [1], which is severely affected by many factors, such as oral hygiene, saliva composition, and dietary carbohydrates [2]. In general, bacteria adhered to the surface of a tooth easily aggregates and secretes a thick matrix of exopolymers that intimately covers and firmly protects bacterial colonies in the manner of a biofilm, thus rendering the bacteria more resistant under ambient conditions [3,4]. In recent years, much research on dental antibacterial resins or adhesives has been conducted [5,6]. For example, in 2021, Choi et al. [7] reported that a Zwitterionic polymer-based coating exhibited superior hydration and anti-polyelectrolyte effect with a reduction of 85% and 80% in ex vivo and in vivo biofilm formation, respectively. In addition, several groups have paid attention to the addition of silver (Ag) nanoparticle (NP) or quaternary ammonium salt into the substrate in the material fabrication due to their excellent antibacterial property [3,4,8,9]. However, the biofilm-based Clear Overlay Appliance (COA) fabricated by these strategies (i) is easily cracked and has to be frequently changed, (ii) suffers from strong adhesion which leads to the severe aggregation of bacteria and dust on the surface, and (iii) the leakage of antibacterial agents causes an unavoidable cytotoxicity [10,11,12]. Therefore, it is necessary to develop a novel dental retainer with low-cost and no-toxicity or low toxicity for excellently bacterial activity.

Artificial self-cleaning materials are capable of creating an extremely water repellent surface, where water droplets enable the formation of approximately spherical shapes to pick up and remove bacteria, dust, and even viruses in the process of rolling, which hold great potentials in the remarkable reduction of bacterial adhesion on the substrate surface [13,14,15,16]. Until now, many examples have demonstrated that self-cleaning surfaces with a contact angle above 90° play vital roles in antifouling and antibacterial applications [17,18,19,20,21]. For instance, in 2022, Narain et al. [18] developed a sugar-responsive and self-cleaning surface with a dual-functional property which not only greatly reduced the bacteria adhesion but also largely promoted the formation of a hydrophilic surface with excellent biocompatibility. Moreover, in 2021, it was reported that the introduction of polydimethylsiloxane (PDMS) could not only largely lower the surface energy of substrates for a self-cleaning function but also significantly enhance their mechanical and chemical stability for practical applications [20]. However, few reports have focused on the hydrophobic surface to remarkably reduce antibacterial adhesion for dental applications so far.

The past decades have witnessed a rapid development in monoelemental Xenes, such as graphdiyne, antimonene, bismuthene, and tellurene, which are promising materials in a variety of fields, including applications in antibacterial activity, optoelectronic devices, photothermal therapy, photocatalysis, energy storage, and multifunctional systems [22,23,24,25,26,27,28,29]. In recent years, Bi nanostructures have drawn extensive attention due to their fascinating properties, such as a high surface area, easy functionalization, narrow bandgap, high X-ray attenuation coefficient, low toxicity, and high stability [30,31,32,33]. It was demonstrated that Bi nanostructures, such as Bi nanoparticles (NPs) [34], and mesoporous silica supported Ag-Bi NPs [35,36], exhibited superior antibacterial performance without antibiotics due to their facile controllability, poor drug resistance, and negligible side effects. The above-mentioned features merit Bi nanostructures as ideal candidates for cost-efficient, stable, and non-toxic antibacterial agents.

In this contribution, zero-dimensional (0D) Bi quantum dots (QDs) were for the first time incorporated into a polymer matrix, PDMS, for dental applications. The 0D Bi QDs with an average diameter of 16 nm and an average thickness of 13 nm were successfully synthesized by a facile solvothermal method. With regard to the high hydrophobicity of PDMS, the PDMS solution was used to evenly distribute Bi QDs to obtain uniform Bi QD/PDMS nanocomposites, which were directly dropped onto the surface of the pristine tooth to form a stable surface after being cured for a predetermined time. The result demonstrates that the as-obtained Bi QD/PDMS-modified tooth not only exhibited a superior hydrophobic behavior, but also displayed an excellent antibacterial activity. Moreover, due to the excellent photothermal effect, the antibacterial activity of the Bi QD/PDMS-modified tooth showed a significant improvement under an external illumination, even at a very low power density (12 mW cm^−2^), indicating that external light can largely enhance the efficiency of bacteria-killing. In addition, the Bi QD/PDMS-modified tooth exhibited a low cell toxicity towards periodontal ligament fibroblasts and periodontal ligament stem cells. Because of the facile synthesis, superior self-cleaning behavior, excellently antibacterial activity and highly photothermal effect, and low cell toxicity, it is envisioned that the Bi nanostructure-based self-cleaning material can provide a fundamental guidance for the antibacterial activity in the field of dentistry, and offer versatile opportunities to exploit high-performance nanostructure-based heterostructures for highly efficient, cost-effective, and smart dental biomaterials.

## 2. Materials and Methods

### 2.1. Materials

Bismuth neodecanoate (technical grade) and 1-octadecene (90%) were purchased from Sigma-Aldrich, Shanghai, China and used upon receipt. 1-dodecanethiol (DDT, 98%) was purchased from Macklin chemistry Co., Ltd., Shanghai, China, and used as received. Tri-n-octylphosphine (TOP, 90%), toluene (99.9%), acetone (99.9%), tetrahydrofuran (THF, 99.9%), potassium chloride (KCl, 99.9%), hydrochloric acid (HCl, 99.9%), and potassium hydroxide (KOH, 99.9%) were purchased from Aladdin chemistry Co., Ltd., Shanghai, China, and used as received. Hydroxyl-terminated PDMS (Sylgard 184A) and a curing agent (Sylgard 184B) were supplied by Dow Corning Corporation, Hangzhou, China. Human periodontal ligament fibroblasts were purchase from Procell Life Sci & Tech Co., Ltd., Wuhan, Hubei, China. Calcein-AM/PI Double Stain kit cell staining agent was purchased from YESEN Biotechnology Co., Ltd., Shanghai, China. The cultivation procedure of periodontal ligament stem cells was carried out according to the previous research methods in our group [37,38] and was approved by the ethics committee of the Affiliated Hospital of Nantong University, Nantong, China. *S. mutans* (ATCC25175) was purchased from Shanghai Bioresource Collection Center, Shanghai, China. Orthodontic premolars from patients aged 14–22 years were collected from the outpatient clinic of the Department of Dentistry, Affiliated Hospital of Nantong University, Nantong, China, after obtaining informed consent from all patients and parents of adolescents. The extracted human molars with complete surfaces were selected and cleaned for standby.

### 2.2. Synthesis and Characterization of 0D Bi QDs

The uniform Bi QDs were synthesized according to Son’s previous reports. Typically, 0.723 g of bismuth neodecanoate (1 mmol) was added to 5 mL of 1-octadecene. The mixture solution was degassed at 120 ℃ for 2.5 h in a vacuum to remove water and oxygen. The solution was then heated to 80 ℃ with vigorous stirring, followed by the addition of 0.24 mL of DDT, and maintained at this temperature for 10 min. After the addition of DDT, the initially colorless solution turned yellow, indicating the formation of bismuth dodecanethiolate complex. Afterwards, the bismuth dodecanethiolate complex solution was cooled to growth temperature, and then 1 mL of TOP was injected into the solution and continued to age at this temperature. The Bi QDs were precipitated by the addition of a 10:1 (*v*/*v*) mixture of acetone and THF, then retrieved by centrifugation, and washed for several times with a 10:1 (*v*/*v*) mixture of acetone and THF. Finally, Bi QDs were redispersed in common nonpolar solvents such as toluene to form long-term stable colloidal dispersions.

The morphology and dimension of the as-synthesized Bi QDs were determined by both transmission electron microscopy (TEM, FEI Tecnai G2 F30, Hillsboro, OR, USA) and atomic force microscopy (AFM, Bruker, with 512 pixels per line, Beijing, China). High-resolution transmission electron microscopy (HRTEM, Hillsboro, OR, USA) was also conducted to determine the atomic arrangement. A high-resolution confocal Raman microscope (HORIBA LabRAM HR800, Paris, France) was performed to record the Raman spectra at room temperature under an excitation wavelength of 633 nm. UV-Vis-NIR absorption spectroscopy was recorded in the spectral range of 200–1000 nm using a UV-Vis absorbance spectrometer (Cary 60, Agilent, Beijing, China).

### 2.3. Preparation of Bi QD/PDMS-Modified Tooth

The suspension containing the mixture of Bi QDs and PDMS (Sylgard 184A: Sylgard 184B = 10: 1) with a Bi QD concentration of 0, 200, 400, or 800 ppm was directly dropped onto the surface of the pristine tooth. The pristine tooth was completely covered by the excess Bi QD/PDMS mixture. Afterwards, the Bi QD/PDMS-coated tooth was cured at a predetermined temperature (27 °C, 37 °C, 47 °C, 57 °C, and 67 °C) for a certain time (30 min, 60 min, 90 min, 120 min, 150 min, 180 min, and 210 min) to obtain a Bi QD/PDMS-modified tooth. Here, the Bi QD/PDMS-modified tooth with a Bi QD concentration of 0 ppm is abbreviated as PDMS-modified tooth.

### 2.4. Hydrophobicity Evaluation

The water contact angle measurements were performed by an optical contact angle measuring instrument (JGW-360B, Beijing, China) at room temperature. A total of 5 μL droplets were dropped onto tooth root and tooth crown for measurement. Each experiment was repeated 3 times.

### 2.5. Antibacterial Activity Evaluation

In order to evaluate the efficiency of the as-fabricated Bi QD/PDMS nanocomposite against cariogenic bacteria, *S. mutans* was chosen, and a direct contact test was used to evaluate the antibacterial activity. The Bi QD/PDMS-modified tooth with different concentrations (0, 200, 400, or 800 ppm) of Bi QDs in the Bi QD/PDMS nanocomposite were placed into a disc with a diameter of 8 mm and thickness of 0.5 mm and the disc was placed in deionized water for 1 h in order to remove free Bi QDs on the surface. After being sterilized by ultraviolet illumination for 2 h, 10 μL suspension containing *S. mutans* (~10^6^ CFU mL^−1^) was poured on the surface of each disc. For better contact between the Bi QD/PDMS nanocomposite and suspension, a polyethylene film was used to gently cover the suspension surface on the tooth. After anaerobic incubation for 24 h, both the discs and PE film were put into a test tube containing 10 mL of axenic physiological saline where the bacteria were totally eluted by shaking and collected. Afterwards, 100 μL of the diluted eluent was transferred on an agar plate for 24 h anaerobic incubation, the groups with a quantity of bacterial colony between 30 to 300 in each gradient were chosen to calculate the quantity of original live bacteria in the discs. The control group was also set under the same conditions in the absence of the Bi QD/PDMS-modified tooth. The entire experiment was repeated 3 times.

### 2.6. Cytotoxicity Analysis

Data collection on cell viability on contact with the surface of the modified tooth was performed by a fluorescent microscope (Olympus, IX73). Cell viability and proliferation was tested using Cell Counting Kit-8 (CCK-8, Beyotime, China) according to the manufacturer’s instruction. Then, 10 uL CCK-8 was added per well in 96-well plates and incubated at 37 °C for 2 h. The optical density was measured at 450 nm. The optical density (OD) value was detected to acquire the cell survival rate of each group using Equation (1)

Cell viability = (OD_e_ − OD_b_)/(OD_c_ − OD_b_) (1)

where OD_e_, OD_b_ and OD_c_ denote the OD value of the experimental group, blank group, and control group, respectively. Cell live/dead staining was performed to investigate the cytotoxicity of the modified tooth using Calcein-AM/PI Double Stain kit. Periodontal ligament fibroblasts were cultured on a Bi QD/PDMS disc for 24 h and collected by centrifugation at 1000 rpm for 5 min. The cells were stained with 2 μM calcein-AM (live cells, green fluorescence) and 4.5 μM PI (dead cells, red fluorescence) for 15 min at 37 °C in the dark.

## 3. Results

**Figure 1:**
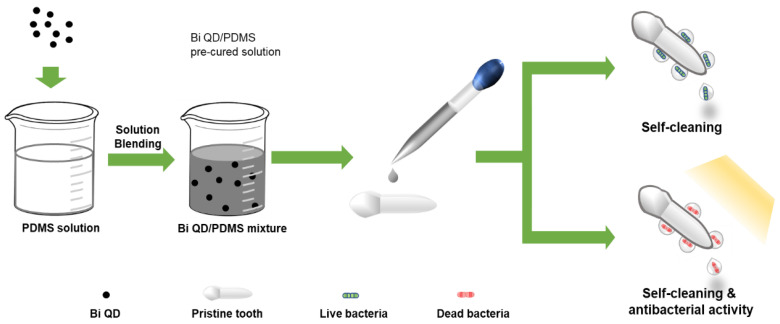
The schematic diagram of the Bi QD/PDMS-modified tooth with both self-cleaning and antibacterial activity.

**Figure 2:**
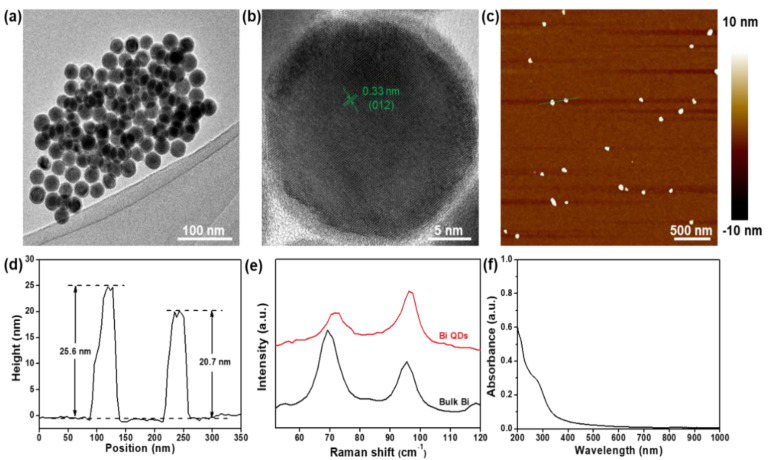
Structural characterization. (**a**) TEM image and (**b**) HRTEM image of the as-synthesized 0D Bi QDs, (**c**) AFM image and (**d**) the corresponding height profile of the 0D Bi QDs, (**e**) Raman spectra of bulk Bi and 0D Bi QDs, and (**f**) UV-Vis-NIR spectrum.

The schematic diagram of the Bi QD/PDMS-modified tooth with both self-cleaning and antibacterial activity is presented in Figure 1. Figure 2 presents the structural characterization of the as-synthesized 0D Bi QDs. The TEM image shows that the Bi QDs were very uniform with an average diameter of 28.6 nm (Figure 2a). The HRTEM image exhibits a clear lattice fringe of 0.33 nm (Figure 2b), which can be indexed to the (012) plane of the Bi crystal [33]. The AFM image presents the typical morphology of the 0D Bi QDs with a uniform distribution (Figure 2c) and the corresponding heights were measured to be 25.6 nm and 20.7 nm (Figure 2d). Raman spectroscopy was also employed to study the as-synthesized 0D Bi QDs, as seen in Figure 2e. The main peaks at 69.4 and 95.6 cm^−1^, can be assigned to the E_g_ and A_1g_ first-order Raman modes of Bi crystal, respectively [22,24]. The peak intensity ratio of A_1g_/E_g_ increased with the decrease in the layer number, i.e., the Bi QDs showed a significantly increased A_1g_/E_g_ value, which is consistent with the previously reported Bi nanostructures [24,28]. Moreover, the as-synthesized 0D Bi QDs displayed a broadband absorption from 200 nm to 650 nm (Figure 2f), which indicates that they have great potential for UV-Vis photoresponsive applications.

**Figure 3:**
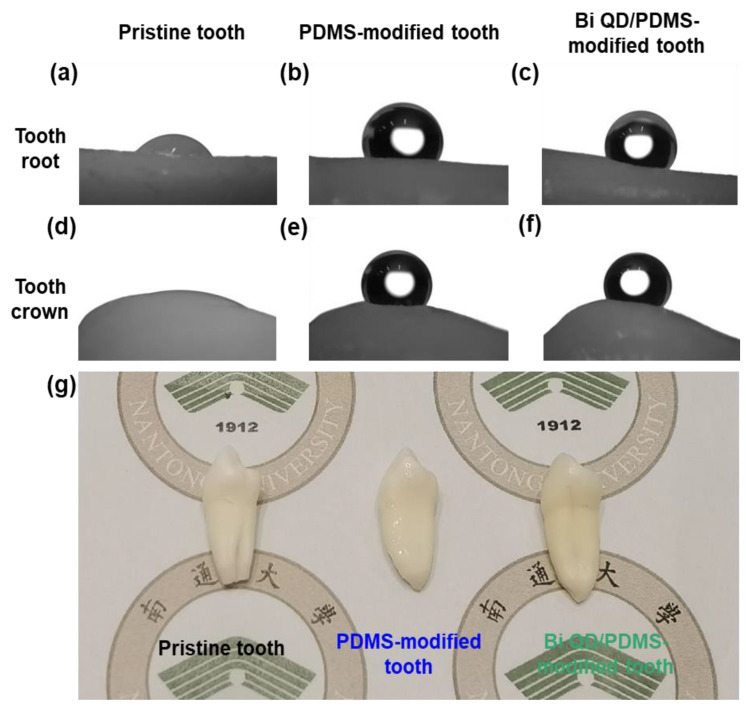
The change of *θ* and morphology after the modification of pristine tooth. The pictures for *θ* on the tooth root for (**a**) pristine tooth, (**b**) PDMS-modified tooth and (**c**) Bi QD/PDMS-modified tooth; the pictures for *θ* on the tooth crown for (**d**) pristine tooth, (**e**) PDMS-modified tooth and (**f**) Bi QD/PDMS-modified tooth. (**g**) The picture for real pristine tooth and modified tooth.

The Bi QD/PDMS nanocomposites were fabricated by a simple solution blending, then directly deposited onto the surface of a clean pristine tooth, and cured for a predetermined time at certain temperature to obtain a Bi QD/PDMS-modified tooth. Notably, regardless of whether the droplet was on the tooth root or tooth crown, the contact angle was remarkably enhanced after the modification by PDMS (Figure 3a,b,d,e), and this was attributed to the excellent hydrophobicity of PDMS [20]. It is noted that PDMS in the Bi QD/PDMS nanocomposites can not only greatly lower the surface energy but also maintain the chemical and mechanical stability after the curing process and have no shrinkage because no monomers are involved to polymerize. Besides, it can be observed that the contact angles for the Bi QD/PDMS-modified tooth with 400 ppm Bi QDs in the Bi QD/PDMS nanocomposite (Figure 3c,f) are well-maintained compared to those of the PDMS-modified tooth (Figure 3d,e), verifying that a tiny amount of 0D Bi QDs have no obvious effect on the hydrophobicity of the tooth studied. Besides, the SEM image confirms that there was a clear polymer aggregation observed (Figure S1). Furthermore, the optical images of the pristine tooth and modified teeth in Figure 3g display that there was no severe aesthetic effect for the modified teeth.

**Figure 4:**
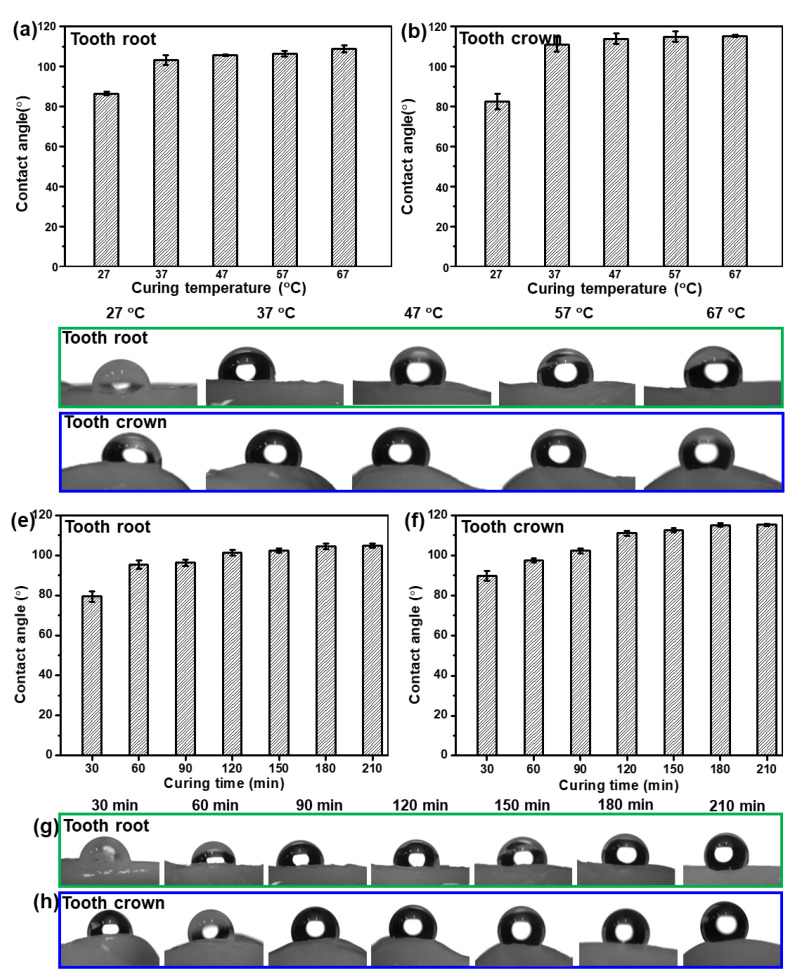
The influence of curing time and temperature on the hydrophobic performance of the as-fabricated Bi QDs/PDMS nanocomposites on the tooth. The *θ* on the (**a**) tooth root and (**b**) tooth crown as a function of curing temperature, and pictures for *θ* on the (**c**) tooth root and (**d**) tooth crown at different curing temperatures for 120 min. The *θ* on the (**e**) tooth root and (**f**) tooth crown as a function of curing time, and pictures for *θ* on the (**g**) tooth root and (**h**) tooth crown at different curing times at the curing temperature of 37 °C.

In order to study the effect of curing time and curing temperature on the hydrophobicity of the as-fabricated Bi QD/PDMS nanocomposites, the Bi QD/PDMS mixture was directly dropped onto a pristine tooth and cured for a predetermined time at a certain temperature. The water contact angle (*θ*) on the tooth root and tooth crown for the as-fabricated Bi QD/PDMS nanocomposites cured for 120 min at different curing temperature is presented, as seen in Figure 4a–d. It can be observed that both the *θ*s on the tooth root and tooth crown remarkably increased when the curing temperature increased from 27 °C to 37 °C, i.e., the average *θ*s on the tooth root and crown at a curing temperature of 27 °C are 86.5° and 82.6°, respectively, while they reached up to 103° and 111°, respectively (Figure 4a,b), revealing the excellent in-air hydrophobicity. However, as the curing temperature further increased, both the *θ*s on the tooth root and tooth crown keep relatively stable (Figure 4a,b), illustrating that the curing temperature of 37 °C is sufficient to make the formation of the hydrophobic Bi QD/PDMS nanocomposites on a pristine tooth. Figure 4c,d also presents the pictures for *θ* on the tooth root and tooth crown at different curing temperatures for 120 min, respectively. In addition, the influence of curing time on the hydrophobicity of the as-fabricated Bi QD/PDMS nanocomposites at a curing temperature of 37 °C can be seen in Figure 4e–h. Figure 4e,f shows that both the *θ*s on the tooth root and tooth crown increased with the curing time prolonged from 30 min to 120 min, yet both of them reached an equilibrium state as the curing time further increased, indicating that the curing process for 120 min at 37 °C is essential for the formation of Bi QD/PDMS nanocomposites on a pristine tooth with excellent hydrophobicity. The pictures for *θ* on the tooth root and tooth crown at different curing times at the curing temperature of 37 °C can be seen in Figure 4g,h, respectively. Moreover, the optical view of the as-fabricated Bi QD/PDMS nanocomposites with different concentrations of Bi QDs in Figure S2a shows that the color of the as-fabricated Bi QD/PDMS nanocomposites became slightly darker with the concentration of Bi QDs. Common liquids, such as water, milk, and coffee, can be sufficiently repelled by the as-fabricated Bi QD/PDMS nanocomposites (Figure S2b), due to the low surface energy caused by PDMS. Moreover, the aesthetic of the PDMS-modified tooth maintains very well compared to that of pristine tooth due to the intimate contact between the pristine tooth and PDMS. The result indicates that this Bi QD/PDMS nanocomposite can shed new light on new designs of the nanostructure/polymer matrix nanocomposites at low temperatures for high-performance self-cleaning dental materials.

**Figure 5:**
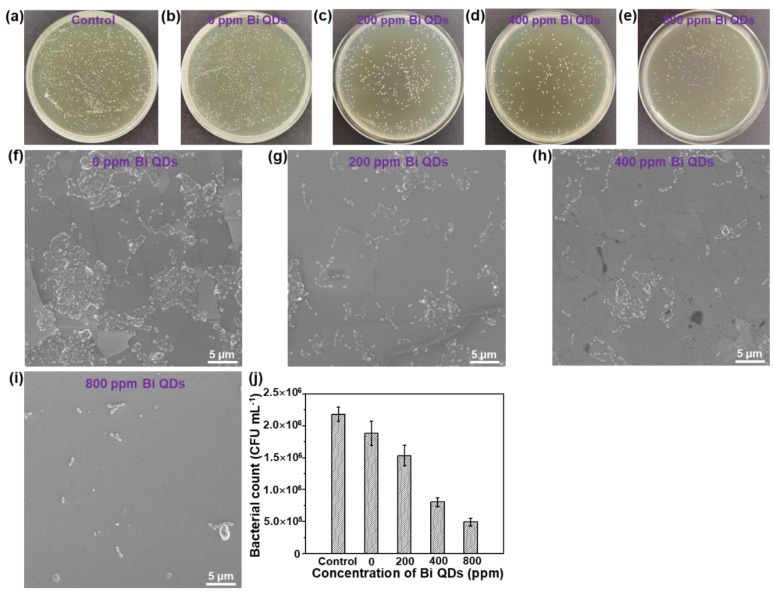
The influence of the Bi QDs content in the Bi QD/PDMS nanocomposite on the antibacterial performance. Plate photographs of *S. mutans* after incubated for 24 h under dark: (**a**) control, (**b**) 0 ppm Bi QDs, (**c**) 200 ppm Bi QDs, (**d**) 400 ppm Bi QDs, and (**e**) 800 ppm. SEM image of *S. mutans* under dark: (**f)** control, (**g**) 0 ppm Bi QDs, (**h**) 200 ppm Bi QDs, (**i**) 400 ppm Bi QDs, and (**j**) 800 ppm. (**k**) Data statistics from the SEM image.

The disk diffusion measurement of the pure 0D Bi QDs with different concentrations can be seen in Figure S3 and Table S1. The result shows that the mean diameter (*d*) distinctly increased with the concentration of the pure 0D Bi QDs, demonstrating that the pure 0D Bi QDs indeed have high antibacterial activity, similar to Ag nanoparticles (NPs) [39], N-doped TiO_2_ NPs [40], and Ca-doped SiO_2_ NPs [41], and can further improve the antibacterial efficiency under light illumination due to the excellent photothermal effect [22,24,42]. The antimicrobial activity of the Bi QD/PDMS nanocomposites with different concentrations of 0D Bi QDs on the *S. mutans* culture is shown in Figure 5. It can be seen that in the control solution without a pristine tooth, PDMS and Bi QDs displayed a rapid growth of *S. mutans* in the dark, and even when diluted 10 times, bacterial colonies were still clearly observed (Figure 5a). The individual cultured bacterial colony is presented in Figure S3. More importantly, the bacterial colonies gradually decreased with the increase in the Bi QDs in the Bi QDs/PDMS nanocomposites (Figure 5b–e), indicating that the Bi QDs in the Bi QD/PDMS nanocomposites on the surface of a pristine tooth can play a vital role in the antibacterial activity in dental applications. Additionally, the cultured bacterial colonies were also characterized by the SEM technique, as shown in Figure 5f–i. The result shows the same trend as that tested by the agar plates. The detailed change of the cultured bacterial colonies can be also seen in Figure 5j. Therefore, the present work developed a new kind of nanocomposites without polymerization shrinkage, which can greatly reduce the bacterial adhesion due to the excellent hydrophobicity of PDMS, and significantly inhibit the biofilm growth due to antibacterial activity.

**Figure 6:**
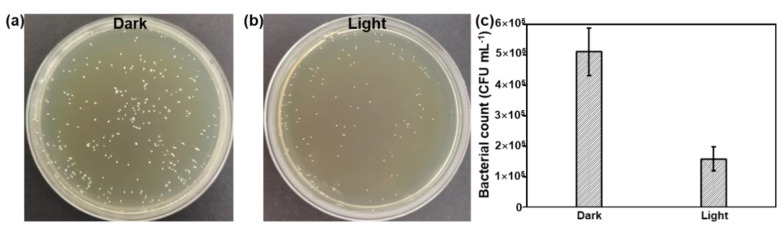
The antibacterial performance of the Bi QD/PDMS-modified tooth. Plate photographs of *S. mutans* for the Bi QD/PDMS-modified tooth incubated for 24 h (**a**) in the dark and (**b**) under light with a power density of 12 mW cm^−2^. (**c**) The comparison of the antibacterial performance for the Bi QD/PDMS-modified tooth with 400 ppm Bi QDs in the Bi QD/PDMS nanocomposite.

To further improve the antibacterial activity of the Bi QD/PDMS nanocomposites, the agar dilution method against *S. mutans* was conducted under dark and light conditions, as shown in Figure 6. Due to the excellent photothermal effect of 0D Bi QDs, [22,24,42] the antibacterial activity of the Bi QD/PDMS nanocomposite with 400 ppm Bi QDs significantly improved (Figure 6a,b), i.e., the bacterial count was reduces to 1.58 × 10^5^ CFU mL^−1^ after being treated under light with an extreme low power density of 12 mW cm^−2^, while it is 5.11 × 10^5^ CFU mL^−1^ conducted in the dark (Figure 6c), suggesting that the 0D Bi QDs in the Bi QD/PDMS nanocomposites can greatly facilitate the antibacterial activity. Moreover, the Bi QD/PDMS nanocomposites also present excellently antibacterial stability even after one month storage (Figure S4). Based on this, the dental materials with multifunctionalities, such as photothermal effect, drug delivery, and drug control release can be rationally designed and experimentally achieved in the next scenario. In addition, the same trend is observed by the agar plate tests as that of the pure Bi QDs, confirmed by SEM measurement Figure S5), verifying that the antibacterial activity of Bi QDs was not significantly affected after the introduction of PDMS, similar to those of resin-based composites, such as core-shell chlorhexidine/amorphous calcium phosphate NP-incorporated resin composites [43], glass-ionomer- incorporated resin composites [44], and nano ZnO-incorporated resin composites [45].

**Figure 7:**
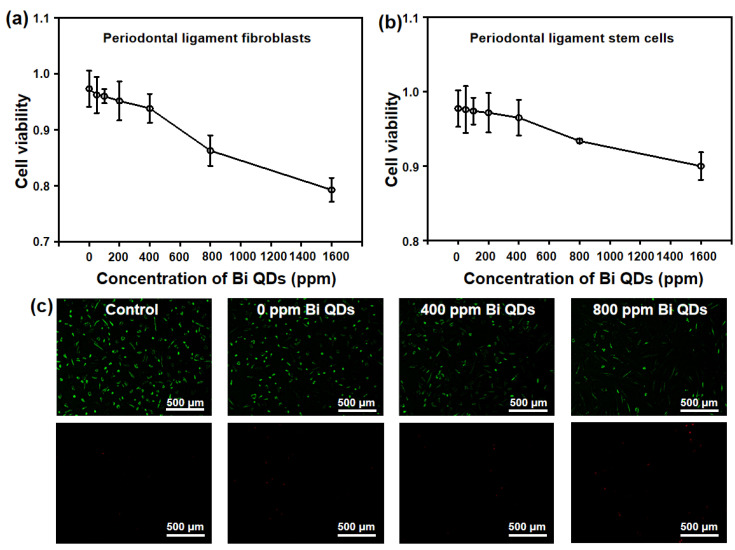
Cytotoxicity of the Bi QD/PDMS-modified tooth with different concentrations of Bi QDs toward (**a**) periodontal ligament fibroblasts and (**b**) periodontal ligament stem cells. The two curves are normalized based on the control groups. (**c**) Live/dead fluorescence images for the periodontal ligament fibroblast cell viability treated by Bi QD/PDMS-modified tooth with different concentrations of Bi QDs for 24 h in the dark. The periodontal ligament fibroblast cells were stained by Calcein-AM/PI Double Stain kit.

Considering the damage to human cells for the Bi QD/PDMS nanocomposites, two main cells in the dental field, periodontal ligament fibroblasts and periodontal ligament stem cells, were selected to study the effect of the concentration of Bi QDs in the Bi QD/PDMS nanocomposite on the cytotoxicity, as shown in Figure 7. It can be seen in Figure 7a,b that the cytotoxicity of Bi QD/PDMS nanocomposites for both of periodontal ligament fibroblasts and periodontal ligament stem cells increased with the increasing concentration of Bi QDs in the Bi QD/PDMS nanocomposites. It should be noted that when the concentration of Bi QDs in the Bi QD/PDMS nanocomposites reached up to 800 ppm, most of the cells keep their vitality, i.e., when the concentration of Bi QDs was 800 ppm, the average optical density (OD) of the periodontal ligament fibroblasts and periodontal ligament stem cells were 0.863 and 0.933, respectively. Moreover, the fluorescence images for the periodontal ligament fibroblast cell viability treated by the Bi QD/PDMS-modified tooth with different concentrations of 0D Bi QDs are also presented, as shown in Figure 7c. Note that there are negligible dead cells observed for the Bi QD/PDMS nanocomposite with 400 ppm Bi QDs, while dead cells can be seen when the concentration of Bi QDs is 800 ppm, indicating that the as-fabricated Bi QD/PDMS nanocomposites hold great promise in practical dental applications.

## 4. Conclusions

In this contribution, the Bi QD/PDMS nanocomposites with both excellent antibacterial activity and hydrophobicity were rationally designed and elaborately synthesized by tuning the curing temperature and time of PDMS. The water contact angle of the new nanocomposites on both the tooth root and crown remarkably increased in the existence of the cured PDMS, showing excellent hydrophobicity for self-cleaning behavior towards common liquids. The antibacterial activity of the Bi QD/PDMS nanocomposites displayed an apparent reduced CFU count with the increasing concentration of Bi QDs. Moreover, the antibacterial activity could be largely enhanced under light illumination even at an extremely low power density (12 mW cm^−2^), due to the photothermal effect of Bi QDs. Additionally, the cytotoxicity of the Bi QD/PDMS-modified tooth demonstrates that there was no severe damage to the normal cells. It is envisioned that the novel Bi QD/PDMS nanocomposite can pave the way to new designs for high-performance nanostructures with multifunctionalities to efficiently protect dental health in daily life.

## Data Availability

The data presented in this study are available on request from the corresponding author.

## Supplementary Materials

The following supporting information can be downloaded at: https://www.mdpi.com/article/10.3390/nano12213911/s1, Figure S1: SEM image of the surface of the as-fabricated Bi QD/PDMS-modified tooth with 400 ppm Bi QDs in the Bi QD/PDMS nanocomposite; Figure S2: (a) The optical images of the as-fabricated Bi QD/PDMS nanocomposites with different concentration of Bi QDs, and (b) the optical images of various liquid droplets setting on the as-fabricated Bi QD/PDMS nanocomposites; Figure S3: Inhibition halo of the Bi QDs against *Streptococcus mutans* strain NG8; Figure S4: The antibacterial performance of the Bi QD/PDMS-modified tooth after one month storage. Plate photographs of *S. mutans* for the Bi QD/PDMS-modified tooth incubated for 24 h (a) in dark and (b) under light with a power density of 12 mW cm^−2^. (c) The comparison of the antibacterial performance for the Bi QD/PDMS-modified tooth with 400 ppm Bi QDs in the Bi QD/PDMS nanocomposite; Figure S5: SEM image of an individual cultured bacterial colony; Table S1: The mean diameters of the inhibition halos of the pure Bi QDs with different concentrations.
