# Supplementary material for: Bismuth Quantum Dot (Bi QD)/Polydimethylsiloxane (PDMS) Nanocomposites with Self-Cleaning and Antibacterial Activity for Dental Applications
Source: Nanomaterials (Basel). 2022 Nov 5;12(21):3911. doi: 10.3390/nano12213911 (PMC9656007; doi:10.3390/nano12213911)
Supplement: Supplementary file 1 [file nanomaterials-12-03911-s001.zip › nanomaterials-1965339-supplementary.pdf]

nanomaterials

MDPI

Supplementary Materials

Bismuth Quantum Dot (Bi QD)/Polydimethylsiloxane (PDMS) Nanocomposites with Self-Cleaning and Antibacterial Activity for Dental Applications

Yingzi Hu [1], Zhiliang Xu [1], Yi Hu [2], Lanping Hu [2], You Zi [2], Mengke Wang [2,*], Xingmei Feng [1,*] and Weichun Huang [2]

[1] Department of Stomatology, Affiliated Hospital of Nantong University, 20 Xisi Road, Nantong 226001, China
[2] School of Chemistry and Chemical Engineering, Nantong University, Nantong 226019, China
* Correspondence: mengkewang@ntu.edu.cn (M.W.); xingmeifeng@ntu.edu.cn (X.F.)

Table S1. The mean diameters of the inhibition halos of the pure Bi QDs with different concentrations.

Concentration of Bi QDs (ppm)	Mean Diameter, *d* (mm)	Δ*d* (mm)
0	5.9	0
62.5	7.0	1.1
125	7.6	1.7
250	7.8	1.9
500	8.1	2.2
1000	9.9	4.0

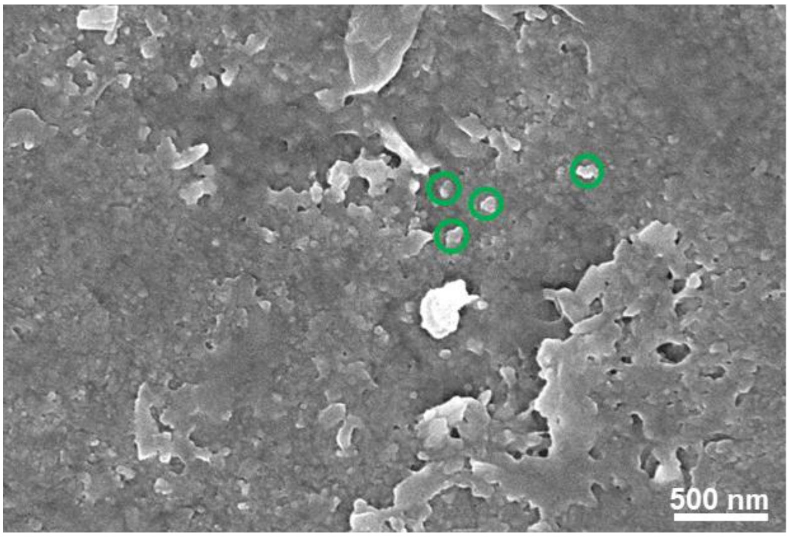

Figure S1. SEM image of the surface of the as-fabricated Bi QD/PDMS-modified tooth with 400 ppm Bi QDs in the Bi QD/PDMS nanocomposite.

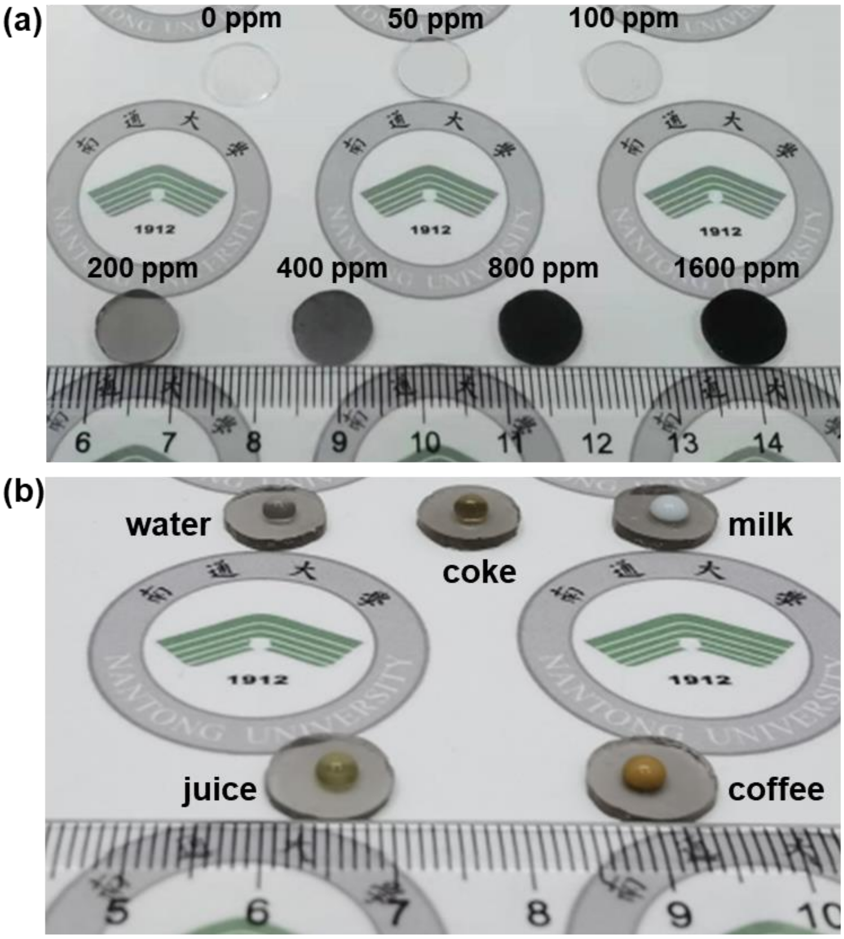

Figure S2. (**a**) The optical images of the as-fabricated Bi QD/PDMS nanocomposites with different concentration of Bi QDs, and (**b**) the optical images of various liquid droplets setting on the as-fabricated Bi QD/PDMS nanocomposites.

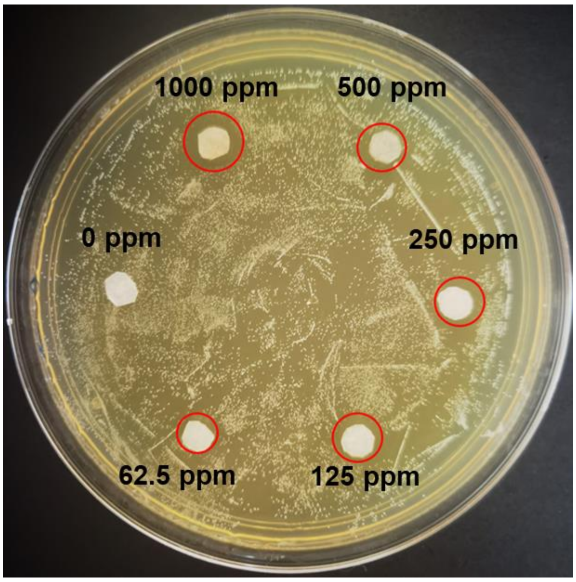

Figure S3. Inhibition halo of the Bi QDs against *Streptococcus mutans* strain NG8.

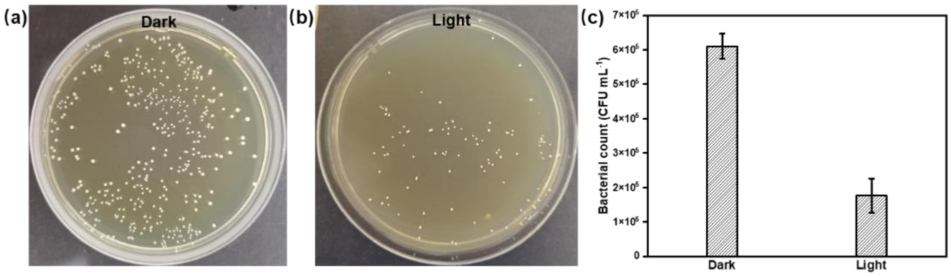

Figure S4. The antibacterial performance of the Bi QD/PDMS-modified tooth after one month storage. Plate photographs of *S. mutans* for the Bi QD/PDMS-modified tooth incubated for 24 h (**a**) in dark and (**b**) under light with a power density of 12 mW cm^{-2}. (**c**) The comparison of the antibacterial performance for the Bi QD/PDMS-modified tooth with 400 ppm Bi QDs in the Bi QD/PDMS nanocomposite.

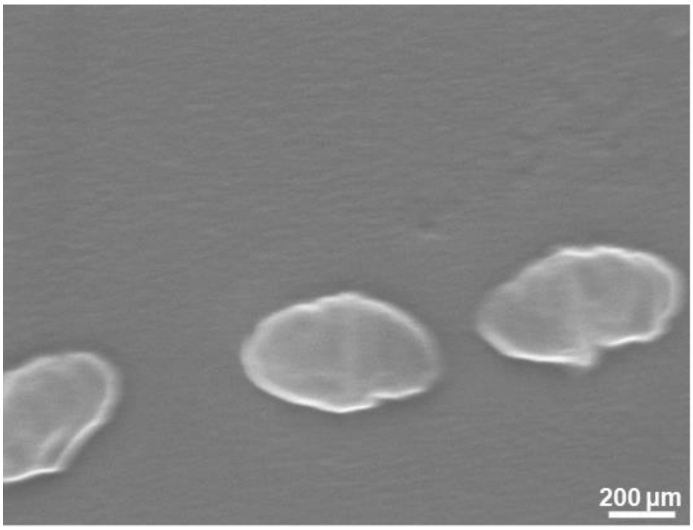

Figure S5. SEM image of an individual cultured bacterial colony.
